# A Multiscale Computational Model Combining a Single Crystal Plasticity Constitutive Model with the Generalized Method of Cells (GMC) for Metallic Polycrystals

**DOI:** 10.3390/ma9050335

**Published:** 2016-05-04

**Authors:** Masoud Ghorbani Moghaddam, Ajit Achuthan, Brett A. Bednarcyk, Steven M. Arnold, Evan J. Pineda

**Affiliations:** 1 Department of Mechanical and Aeronautical Engineering, Clarkson University, Potsdam, NY 13699, USA; ghorbam@clarkson.edu; 2 NASA Glenn Research Center, Cleveland, OH 44135, USA; brett.a.bednarcyk@nasa.gov (B.A.B.); steven.m.arnold@nasa.gov (S.M.A.); evan.j.pineda@nasa.gov (E.J.P.)

**Keywords:** multiscale computational model, metallic polycrystals, Generalized Method of Cells homogenization, crystal plasticity constitutive model

## Abstract

A multiscale computational model is developed for determining the elasto-plastic behavior of polycrystal metals by employing a single crystal plasticity constitutive model that can capture the microstructural scale stress field on a finite element analysis (FEA) framework. The generalized method of cells (GMC) micromechanics model is used for homogenizing the local field quantities. At first, the stand-alone GMC is applied for studying simple material microstructures such as a repeating unit cell (RUC) containing single grain or two grains under uniaxial loading conditions. For verification, the results obtained by the stand-alone GMC are compared to those from an analogous FEA model incorporating the same single crystal plasticity constitutive model. This verification is then extended to samples containing tens to hundreds of grains. The results demonstrate that the GMC homogenization combined with the crystal plasticity constitutive framework is a promising approach for failure analysis of structures as it allows for properly predicting the von Mises stress in the entire RUC, in an average sense, as well as in the local microstructural level, *i.e.*, each individual grain. Two–three orders of saving in computational cost, at the expense of some accuracy in prediction, especially in the prediction of the components of local tensor field quantities and the quantities near the grain boundaries, was obtained with GMC. Finally, the capability of the developed multiscale model linking FEA and GMC to solve real-life-sized structures is demonstrated by successfully analyzing an engine disc component and determining the microstructural scale details of the field quantities.

## 1. Introduction

Engine fan blades and discs are subjected to extreme temperatures and mechanical stresses during their operation. The prediction of damage initiation and propagation is important in these engine components, not only to avoid potential catastrophic mission failures, but for developing optimal and economical designs as well. Developing such prediction capabilities requires development of stress analysis models that can incorporate the interaction of various microstructural features, such as grain boundaries and dislocations.

For metals and metallic alloys, the major deformation mechanism at the microstructural scale is the dislocation motion causing plasticity. Two key characteristics that define plasticity are the permanent strain due to slipping of atomic layers under shear stress and the strain hardening, both contributing to the macroscale nonlinear stress-strain behavior. In the single crystal plasticity models, the dislocation motion is implemented as permanent shear strain in the various slip systems (slip plane and slip direction) with the aid of a set of state variables [1,2]. The strain hardening property that defines the strength of the slip systems is also implemented in terms of another set of state variables. The nonlinear evolution laws are defined for the permanent shear strains and strength of slip systems, along with the equilibrium equations in an incremental and iterative approach.

The major challenge in implementing microstructure-based plasticity models in real-sized structural components is the excessive computational cost, as it requires solving governing equations at both the microstructural and macrostructural length scales. Even for modeling a small subscale volume consisting of tens to a few hundred grains, the computational cost for executing a finite element model incorporating the physics of dislocation at the microscale motion is exorbitant. To address this challenge, the development of various multiscale models, broadly classified under three major methods, namely, sequential (or hierarchical), computational homogenization scheme (so called ${\text{FE}}^{2}$) and integrated technique, were reported [3,4,5,6].

In the sequential method, the macro model utilizes a number of parameters which are either obtained from a detailed microscale experimental analysis or pre-computed [7,8,9,10,11,12,13]. As a result, these models are effectively macroscopic models, but use internal state variables for describing the material behavior at microscale. Therefore, sequential methods are valid for prediction and computationally very efficient. However, the macroscale models are typically calibrated using a finite number of loading scenarios and do not account for the material’s microstructure explicitly. Thus, sequential methods may lack some fidelity in capturing the anisotropic constitutive response of a polycrystal under completely general loading scenarios.

The computational homogenization scheme, on the other hand, can relate the complex material behavior at microscale to its macroscale response by explicitly modeling the material’s microstructure [6,14,15,16,17,18,19,20,21,22]. In these methods, the local governing behavior at the macroscale is determined by solving the microscale boundary value problem on a representative volume element. This also affords the use of simpler, more fundamental constitutive models at the microscale, since the anisotropic nonlinearity at the macroscale arises naturally due to the interaction of the various phases at the microscale. This micro-macro transition can also be implemented by considering coupled boundary value problems at the micro- and macroscales, and solving them simultaneously by means of finite element methods. The computational homogenization scheme, though offering high fidelity, can still be computationally expensive for many practical problems.

The integrated method is based on applying a homogenization technique to an embedded polycrystal in a finite element framework. The homogenization technique, mostly mean-field theories, serves as the transition step between the micro and macro scales [5,23,24,25,26,27]. The microstructure is discretized and split into different phases with analytical relations describing the interaction between the phases. While providing relatively good accuracy, depending on the mean-field theory used, these methods are computationally very efficient. Recent studies have demonstrated successful implementation of a viscoplastic self-consistent model for composite materials, both under explicit [23,24,26] and implicit schemes [25].

A main challenge in the development of a homogenization technique is relating the macroscale average strain at a point to the microstructural representation of the material. The homogenization technique is formulated following two major methods: (1) The Representative Volume Element (RVE) method based on statistical homogenization theories considering the random distribution of the microstructural features; and (2) The Repeating Unit Cells (RUC) method based on periodicity in the microstructure. The definition of RVE requires the equivalent homogenized displacement and traction boundary conditions to be simultaneously satisfied. The RUC-based methods were proposed to address the difficulty in satisfying the required homogenous traction–displacement boundary condition for arbitrarily chosen, statistically homogeneous, microstructures in the RVE concept. Besides analytical methods, those mainly developed for elastic materials, the numerical solutions of the RUC using boundary element method [28], the discrete Fourier transform approach [29,30] and the finite element method [31], have been developed for inelastic, viscoelastic and viscoplastic materials. These RUC-based homogenization techniques are mostly restricted to specific unit-cell architecture, loading direction and simplified boundary condition.

Semi-analytical techniques such as the generalized method of cells (GMC) have also been developed for RUC homogenization [32,33,34,35]. The GMC method enables modeling of complex unit-cell architectures and is capable of studying the elastic–plastic response of periodic heterogeneous materials under various loading conditions. In GMC, an RUC is discretized into a number of sub-volumes, referred to as subcells. Within each of these subcells, the displacement fields are approximated linearly. Traction and displacement continuity conditions are utilized to calculate a strain concentration matrix allowing localization of the applied, average, far-field strains to local subcell strains. The GMC method does not include coupling between the shear and normal components of stress. Many micromechanics theories exhibit this lack of coupling, which typically yields ultra-efficient computational performance. However, retaining this coupling is only critical when this coupling is a first-order effect. Advanced methods [33,34,36,37], such as the High-Fidelity Generalized Method of Cells (HFGMC) were proposed to account for the shear coupling through quadratic displacement approximations in the subcells. However, increase in fidelity incurs a penalty on the computational efficiency.

In this article, GMC is evaluated as a potential method of homogenization to develop a multiscale model that can capture microscale plastic deformation in polycrystal metals and metallic alloys. This work is an extension of a previous study by authors [38,39] on the applicability of GMC homogenization for studying two-phase materials, e.g., Ni-base superalloys, characterized by crystal plasticity framework at microstructures. Polycrystalline materials, with several randomly oriented grains, demonstrate high material anisotropy; this anisotropy introduces its own challenges and is the focus for this study. At first, the performance of the stand-alone GMC for simple test cases, in terms of solution accuracy and computational time, is evaluated by comparing the results with a finite element model that uses the same material subroutine. The polycrystal microstructure is simulated using a pre-processor functionality that creates Voronoi cell tessellations and assigns random orientations. A small polycrystal sample with 8, 27 and 125 grains is then simulated using stand-alone GMC compared with the FEA model. Finally, the multiscale model implemented on a finite element analysis framework at macroscale, with the element properties defined at microscale using GMC homogenization, is evaluated by analyzing a realistic engine disc component.

## 2. Theory & Numerical Implementation

### 2.1. Single Crystal Plasticity Model

The kinematics for the deformation mechanics of crystals follows the pioneering work of Taylor [40], and Hill and Rice [41]. The theory is based on the assumption that any elasto-plastic deformation can be split into two multiplicative operations: a plastic deformation where material is deformed through the rearrangement of lattices, followed by an elastic deformation associated with the stretching of lattices. The total deformation gradient *F* is then given by:

(1) $$F=F^{e}\cdot F^{p}$$

where $F^{p}$ and $F^{e}$ are the plastic and the elastic deformation gradient, respectively.

Based on the deformation gradient definition, the total velocity gradient L is stated as:

(2) $$L=\dot{F}\cdot F^{-1}=D+\Omega$$

In which the symmetric stretch rate D and the anti-symmetric spin tensor Ω can be decomposed into lattice and plastic parts (()*,()^p^), respectively.

(3) $$D=D^{*}+D^{p}\;\Omega =\Omega^{*}+\Omega^{p}$$

The velocity gradient associated with the plastic deformation $L^{p}$ is given in terms of Schmid’s law as:

(4) $$L^{p}=F^{p}\cdot {\dot{F}}^{p}{}^{-1}=\sum_{\alpha }{\dot{\gamma }}^{\alpha }m^{\alpha }⨂n^{a}$$

where ${\dot{\gamma }}^{\alpha }$ is the rate of shear strain associated with the slipping of α slip system; *m* is the unit normal to the slip plane; and *n* is the unit vector parallel to slip direction. The incremental formulation of plasticity theory is based on (1) the evolution of Cauchy stress in the corotational frame of reference that rotates with the crystal lattice, $J^{*}\left(\sigma \right)$; (2) the slipping rate ${\dot{\gamma }}^{\left(\alpha \right)}$ and (3) the strain hardening rate ${\dot{g}}^{\left(\alpha \right)}$ as given below:

(5) $$J^{*}\left(\sigma \right)+\sigma \left(I:D^{*}\right)=C:D^{*}$$

where σ is the Cauchy stress; $D^{*}$ is rate of stretching associated with elastic deformation; and C is the tensor of elastic moduli. The governing equation for slipping is defined as:

(6) $${\dot{\gamma }}^{\left(\alpha \right)}={\dot{a}}^{\left(\alpha \right)}{\left(\frac{\tau^{\alpha }}{g^{\alpha }}\right)}^{n}$$

where $\tau^{\alpha }$ is the resolved shear stress; $g^{\alpha }$ is the strength; the ${\dot{a}}^{\left(\alpha \right)}$ refers to the slipping rate when the resolved shear stress reaches the strength. The governing equation for the strength can be modeled as:

(7) $${\dot{g}}^{\left(\alpha \right)}=\sum_{\beta }h_{\alpha \beta }\left(\gamma \right){\dot{\gamma }}^{\left(\alpha \right)}$$

where $h_{\alpha \beta }$ is the slip-hardening moduli. The sum ranges over all activated slip systems. The coefficient $h_{\alpha \beta }$ represents the self-hardening modulus when α=β, and the latent-hardening modulus otherwise [42].

(8) $$\;h_{\alpha \alpha }=h\left(\gamma \right)=h_{0}sec^{2}\left|\frac{h_{0}\gamma }{\tau_{s}-\tau_{0}}\right|$$

where $h_{0}$ is the initial hardening modulus; $\tau_{0}$ is the yield stress which equals the initial value of current strength $g^{\left(\alpha \right)}$(0); $\tau_{s}$ is the stage-I stress (or the break-through stress where large plastic flow initiates); and γ is the Taylor cumulative shear strain on all slip systems, *i.e.*,

(9) $$\gamma =\sum_{\alpha }\int_{0}^{t}\left|{\dot{\gamma }}^{\left(\alpha \right)}\right|dt$$

The latent hardening modulus is given by:

(10) $$h_{\alpha \beta }=qh\left(\gamma \right)\cdot \left(\alpha \neq \beta \right)$$

where q is a constant. Finally, the instantaneous shear strength of a slip system is then obtained as:

(11) $$g^{\left(\alpha \right)}=\tau_{0}+\int_{0}^{t}{\dot{g}}^{\left(\alpha \right)}\;dt$$

For the numerical implementation of a single crystal plasticity model, a User MATerial (UMAT) subroutine in the form of for Abaqus [43] is used.

### 2.2. Generalized Method of Cells

**Figure 1:**
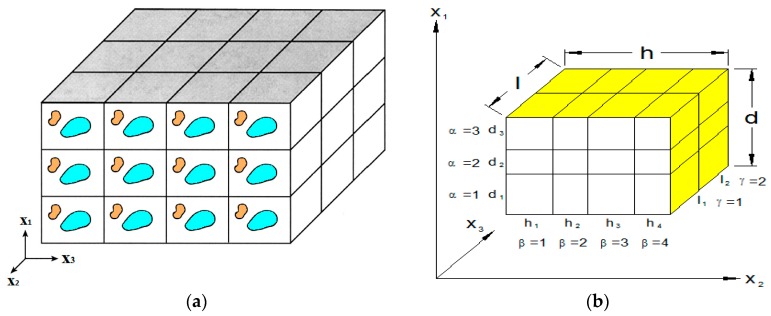
(**a**) Repeating Unit Cells (RUCs); (**b**) A sample RUC discretization consisting of $N_{\alpha }=3$, $N_{\beta }=4$ and $N_{\gamma }=2$ subcells.

The generalized method of cells is a micromechanical formulation for predicting the overall thermo-inelastic behavior of the multiscale composites. There are four steps involved in this homogenization process [33,44]. First, the RUC should be identified (Figure 1a) and discretized. A typical RUC consists of $N_{\alpha }\times N_{\beta }\times N_{\gamma }$ rectangular subcells ($\alpha =1,\ldots ,N_{\alpha };\;\beta =1,\ldots ,N_{\beta };\;\gamma =1,\ldots ,N_{\gamma })$ in the $x_{1},\;x_{2}\;$and $x_{3}$ directions, respectively, as shown in Figure 1b. The individual subcells have the dimensions of $\left(d_{\alpha },h_{\beta },l_{\gamma }\right)$, and are related to the unit cell dimensions(d,h,l) by:

(12) $$d=\sum_{\alpha =1}^{N_{\alpha }}d_{\alpha },\;h=\sum_{\beta =1}^{N_{\beta }}d_{\beta },\;l=\sum_{\gamma =1}^{N_{\gamma }}d_{\gamma }$$

As the second step, the relationships between the macroscopic average stresses and strains with the microscopic fields are established. A linearly variable displacement, $u_{i}^{\left(\alpha \beta \gamma \right)}=w_{i}^{\left(\alpha \beta \gamma \right)}\left(x\right)+{\bar{x}}_{1}^{\left(\alpha \right)}\;\emptyset_{i}^{\left(\alpha \beta \gamma \right)}+{\bar{x}}_{2}^{\left(\beta \right)}\;\chi_{i}^{\left(\alpha \beta \gamma \right)}+{\bar{x}}_{3}^{\left(\gamma \right)}\;\xi_{i}^{\left(\alpha \beta \gamma \right)}\left(i=1,2,3\right)$ is considered inside each subcell. This displacement profile consists of displacement components at the center of each unit cell, $w_{i}^{\left(\alpha \beta \gamma \right)}$, and the microvariables $\emptyset_{i}^{\left(\alpha \beta \gamma \right)}$, $\;\chi_{i}^{\left(\alpha \beta \gamma \right)}$, and $\;\xi_{i}^{\left(\alpha \beta \gamma \right)}$that characterize the linear dependence of the displacement $u_{i}^{\left(\alpha \beta \gamma \right)}$on the local coordinates ${\bar{x}}_{1}^{\left(\alpha \right)}$, ${\bar{x}}_{2}^{\left(\beta \right)}$, and ${\bar{x}}_{3}^{\left(\gamma \right)}$. The vector $x=\left(x_{1},\;x_{2},\;x_{3}\right)$ defines the position of the center of the subcell with respect to the global coordinate system.

The definition of strain tensor in each subcell ${\bar{\varepsilon }}_{ij}^{\left(\alpha \beta \gamma \right)}$ is given as:

(13) $${\bar{\;\varepsilon }}_{ij}^{\left(\alpha \beta \gamma \right)}=\frac{1}{2}\left(\partial_{i}u_{j}^{\left(\alpha \beta \gamma \right)}+\partial_{j}u_{i}^{\left(\alpha \beta \gamma \right)}\right)$$

Then, the average strain in the RUC is defined as:

(14) $${\bar{\varepsilon }}_{ij}=\frac{1}{dhl}\sum_{\alpha =1}^{N_{\alpha }}\sum_{\beta =1}^{N_{\beta }}\sum_{\gamma =1}^{N_{\gamma }}d_{\alpha }h_{\beta }l_{\gamma }{\bar{\varepsilon }}_{ij}^{\left(\alpha \beta \gamma \right)}$$

Considering a general constitutive equation for thermo-elastic-plastic materials in each subcell (α, β, γ), the average stress in that subcell is considered as:

(15) $${\bar{\sigma }}_{ij}^{\left(\alpha \beta \gamma \right)}=C_{ijkl}^{\left(\alpha \beta \gamma \right)}\left({\bar{\varepsilon }}_{kl}^{\left(\alpha \beta \gamma \right)}-{\bar{\varepsilon }}_{kl}^{I\;\left(\alpha \beta \gamma \right)}-{\bar{\varepsilon }}_{kl}^{T\;\left(\alpha \beta \gamma \right)}\right)$$

where the $C_{ijkl}^{\left(\alpha \beta \gamma \right)}$ is the elastic tensor, and the repeated indices ‘k’ and ‘l’ represented a summation following the Einstein notation. The ${\bar{\varepsilon }}_{kl}^{I\;\left(\alpha \beta \gamma \right)}$ and ${\bar{\varepsilon }}_{kl}^{T\;\left(\alpha \beta \gamma \right)}\;$are the average inelastic and thermal strain tensors, respectively, in each subcell.

Based on the volumetric summation of the average stresses in all subcells, the average stress in the entire unit-cell can be written as:

(16) $${\bar{\sigma }}_{ij}=\frac{1}{dhl}\sum_{\alpha =1}^{N_{\alpha }}\sum_{\beta =1}^{N_{\beta }}\sum_{\gamma =1}^{N_{\gamma }}d_{\alpha }h_{\beta }l_{\gamma }{\bar{\sigma }}_{ij}^{\left(\alpha \beta \gamma \right)}$$

In the third step, the continuity of strain and traction is applied. It is considered that the RUCs are periodic, and the interfaces of the subcells and the boundaries of the RUCs follow the displacement and traction continuity. Applying these conditions results in elimination of the microvariables in the displacement definition and produces a set of continuity relationships. As a result, a set of $N_{\alpha }\left(N_{\beta }+N_{\gamma }+1\right)+N_{\beta }\left(N_{\gamma }+1\right)+N_{\gamma }\;$equations stems from the strain continuity relation which can be declared as:

(17) $$A_{G}\varepsilon_{s}=J\bar{\varepsilon }$$

with $A_{G}\;$and J being matrices representing the geometrical details of the subcells and those related to the unit-cell itself, respectively. The $\bar{\varepsilon }$ is the average strain in the unit-cell, while $\varepsilon_{s}$ is defined as:

(18) $$\varepsilon_{s}=\left({\bar{\varepsilon }}^{\left(111\right)},\;\ldots ,\;{\bar{\varepsilon }}^{\left(N_{\alpha },\;N_{\beta },\;N_{\gamma }\;\right)}\right)$$

The continuity of tractions, results in:

(19) $$A_{M}\left(\varepsilon_{s}-\varepsilon_{s}^{I}-\varepsilon_{s}^{T}\right)=0$$

which contains $6N_{\alpha }N_{\beta }N_{\gamma }-\left(N_{\alpha }N_{\beta }+N_{\alpha }N_{\gamma }+N_{\beta }N_{\gamma }\right)-\left(N_{\alpha }+N_{\beta }+N_{\gamma }\right)$ equations, in which $A_{M}$ includes the information regarding the material properties in each subcell. $\varepsilon_{s}^{I}$ and $\varepsilon_{s}^{T}$ represent the inelastic and thermal strains in each subcell, defined similarly to Equation (18).

The strain and traction continuity conditions (Equations (17) and (18)) can be combined as:

(20) $$\tilde{A}\varepsilon_{s}-\tilde{D}\left(\varepsilon_{s}^{I}+\varepsilon_{s}^{T}\right)=K\bar{\varepsilon }\;\text{where},\;\tilde{A}=\left[\begin{array}{c} A_{M}\\ A_{G} \end{array}\right],\;\tilde{D}=\left[\begin{array}{c} A_{M}\\ 0 \end{array}\right],\;K=\left[\begin{array}{c} 0\\ J \end{array}\right]$$

Rewriting this equation for each subcell as:

(21) $${\bar{\varepsilon }}^{\left(\alpha \beta \gamma \right)}=A^{\left(\alpha \beta \gamma \right)}\bar{\varepsilon }+D^{\left(\alpha \beta \gamma \right)}\left(\varepsilon_{s}^{I}+\varepsilon_{s}^{T}\right)$$

allows the average strain in the subcell to be expressed in terms of the macrostrain, and the inelastic and thermal subcell strains by defining the concentration of elastic and inelastic matrices $A^{\left(\alpha \beta \gamma \right)}$ and $D^{\left(\alpha \beta \gamma \right)}$, respectively.

In the fourth step, the overall macroscopic constitutive equations of the material are obtained based on the effective properties. The average (homogenized) constitutive equation for the RUC is:

(22) $$\bar{\sigma }=B^{*}\left(\bar{\varepsilon }-{\bar{\varepsilon }}^{I}-{\bar{\varepsilon }}^{T}\right)$$

in which the $B^{*}$ represents the effective elastic tensor of the polycrystal,

(23) $$B^{*}=\frac{1}{dhl}\sum_{\alpha =1}^{N_{\alpha }}\sum_{\beta =1}^{N_{\beta }}\sum_{\gamma =1}^{N_{\gamma }}d_{\alpha }h_{\beta }l_{\gamma }\;C^{\left(\alpha \beta \gamma \right)}A^{\left(\alpha \beta \gamma \right)}$$

The global inelastic strain tensor ${\bar{\varepsilon }}^{I}\;$and the average thermal strain tensor ${\bar{\varepsilon }}^{T}$ are also obtained in a similar fashion.

(24) $${\bar{\varepsilon }}^{I}=\frac{-B^{*}{}^{-1}}{dhl}\sum_{\alpha =1}^{N_{\alpha }}\sum_{\beta =1}^{N_{\beta }}\sum_{\gamma =1}^{N_{\gamma }}d_{\alpha }h_{\beta }l_{\gamma }\;C^{\left(\alpha \beta \gamma \right)}\left(D^{\left(\alpha \beta \gamma \right)}\varepsilon_{s}^{I}-{\bar{\varepsilon }}^{I\left(\alpha \beta \gamma \right)}\right)$$

(25) $${\bar{\varepsilon }}^{T}=\frac{-B^{*}{}^{-1}}{dhl}\sum_{\alpha =1}^{N_{\alpha }}\sum_{\beta =1}^{N_{\beta }}\sum_{\gamma =1}^{N_{\gamma }}d_{\alpha }h_{\beta }l_{\gamma }\;C^{\left(\alpha \beta \gamma \right)}\left(D^{\left(\alpha \beta \gamma \right)}\varepsilon_{s}^{T}-{\bar{\varepsilon }}^{T\left(\alpha \beta \gamma \right)}\right)$$

It should be noted that GMC was reformulated to solve for subcell tractions, as opposed to strains, to improve computational efficiency [35].

### 2.3. MAC/GMC

The Micromechanics Analysis Code with Generalized Method of Cells (MAC/GMC), developed by NASA Glenn Research Center [45], which predicts the effective nonlinear response of heterogeneous materials based on the known behavior of the constituent materials, is used in the present study. The MAC/GMC software, like most of the commercially available FEA packages, admits user-defined constitutive models. This capability was used to incorporate the single crystal plasticity model within the code such that the inelastic behavior of the individual constituent materials could be represented. The reformulated GMC [35] is called at finite element level to represent the material response of that element using NASA’s FEAMAC software [43,45]. 

## 3. Results and Discussion

The main objective for a multiscale analysis is to provide an accurate constitutive response to the higher scale, being the average stress and strain behaviors of the microscale. In addition, for a failure study of a structure, it is helpful to be able to predict the field quantities at a microstructural length scale. In the results and discussion section of this study it has been attempted to evaluate the GMC homogenized solution combined with the crystal plasticity framework in polycrystalline materials with regard to both the entire RUC and the local microstructural behaviors.

In order to evaluate GMC for studying the plasticity in polycrystals, several virtual test cases, under a tensile stress of 200 MPa with a constant loading rate of 200 MPa/s applied in *z* direction, were considered. In the FEA model, periodic boundary conditions were applied in all three directions to simulate the periodicity of the unit cell in a bulk domain. At first, the stress–strain behaviors of a single crystal sample under uniaxial loading was analyzed and compared with FEA. This was followed by a polycrystalline sample made up of two grains. Both the global stress–strain curves obtained from the entire domain and the local stress-strain curves of the individual grains were compared. Then, the analyses of polycrystals with 27, 64 and 125 grains were implemented. A face-centered-cubic (FCC) copper sample with 12 active slip systems ({111} <110> family) and the cubic material properties ($c_{11}=168,400\text{ MPa},\;c_{12}=121,400\text{ MPa},\;c_{44}=75,400\text{ MPa}$) are used in this study. For the hardening law, $h_{0}=541.5\text{ MPa},\;\tau_{s}=109.5\text{ MPa}$ and $\tau_{0}=60.8\text{ MPa}$ were applied. In addition, the rate sensitivity exponent n, reference strain rate $\dot{a}$. and the ratio of latent to self-hardening moduli q. were taken as 10 and 0.001 $s^{-1}$ and 1, respectively, for the present study.

The polycrystal grain structure was simulated using the Voronoi tessellations and the grain orientations were randomly assigned using MATLAB built-in functions. The GMC uses a linear displacement approximation in each subcell, and as a result of the lack of normal-shear coupling, as long as the geometry of the phases in the RUC is fixed, the solution of GMC is completely insensitive to refinement of the subcell grid. More discussions on the mesh sensitivity in GMC are available in [33]. However, for similar element-wise resolution in the polycrystalline samples in both the FEA and GMC, to obtain a proper solution in FEA 20-node-brick elements were required.

Finally, the capability of GMC homogenization to solve large-scale problems was demonstrated by using the FEAMAC multiscale model framework. A 254 mm radius turbine disk was analyzed using FEA at the macroscale. At each element integration point, an RUC representing a microstrcuture consisting of 27 grain polycrystal material is used to model the local non-linear material repsonse. The homogenization at the element level is carried out by GMC with 1000 subcells.

### 3.1. Single Crystal Behavior

**Figure 2:**
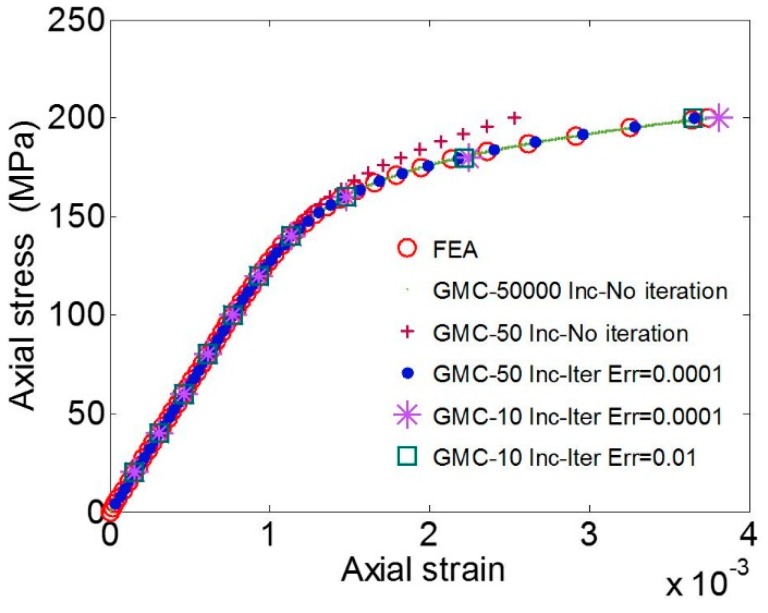
Average axial stress component (*σ_zz_*) *vs*. average axial strain (*ε_zz_*) determined by GMC compared to FEA for single crystal FCC copper (loaded in (1,1,0) direction of the crystal)*.*

A 100 × 10 × 10 ${\text{mm}}^{3}$ single crystal copper sample, with (1,1,0) crystal orientation modeled by 90 subcells/elements under a uniaxial tensile loading in the sample length direction (*z* direction), was considered. The corresponding results in terms of the variation of average axial stress (*σ_zz_*) *vs.* the average axial strain (*ε_zz_*) are shown in Figure 2.

The GMC convergence was studied by varying time increments, number of iterations and permitted error (ERR) for the convergence. It is observed that the GMC solution can converge to the solution from FEA, either by refining the load increments without changing the number of iterations, or, for the smaller number of load increments, by allowing for more iterations.

### 3.2. Polycrystal Behavior

Applicability of the GMC base homogenization approach for polycrystalline samples are studied in this section. At first, in Section 3.2.1, a polycrystalline sample consisting of two grains with maximum possible anisotropy with respect to the grain orientations is analyzed. Then, Voronoi polycrystals with several numbers of grains with randomly oriented grains, representing real material microstructures, are studied in Section 3.2.2.

#### 3.2.1. Two-Grain Polycrystal

**Figure 3:**
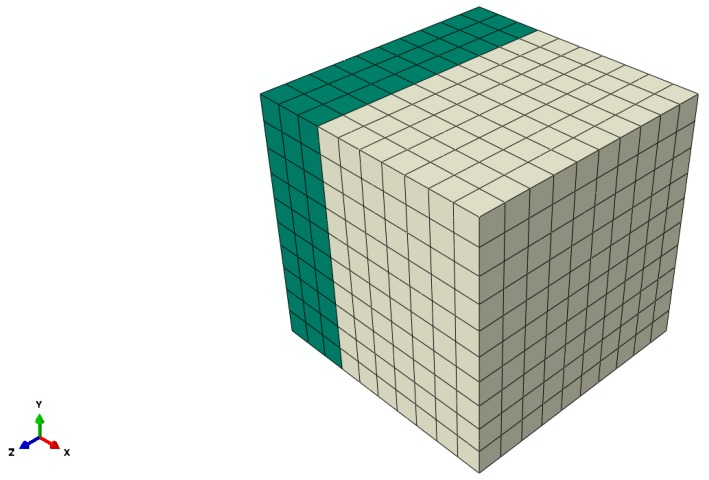
Polycrystal sample consisting of two grains (loaded is in the *z*-direction).

A simple polycrystal consisting of two grains, as shown in Figure 3, is considered. The orientations chosen for the grains are shown in Table 1. The arbitrarily chosen orientation provides a high degree of anisotropy in the studied sample.

**Figure 4:**
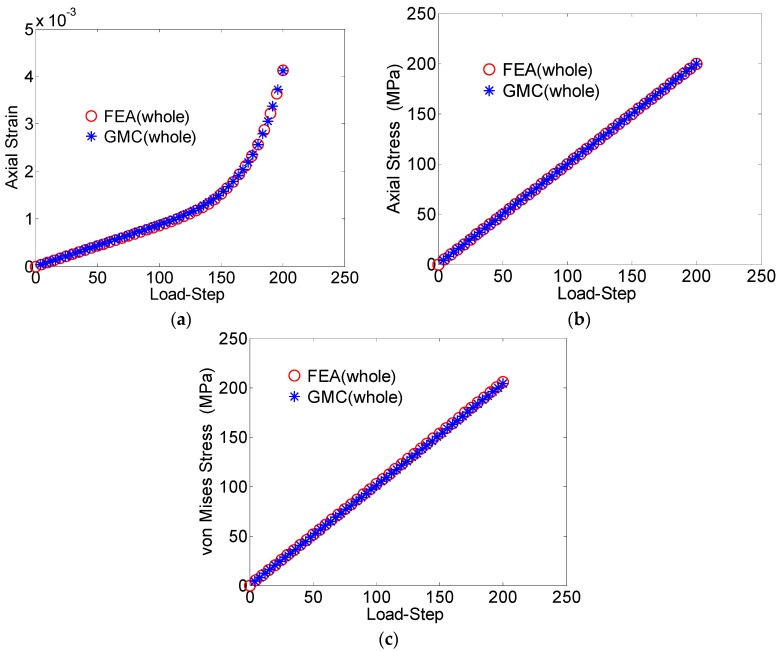
(**a**) Average axial strain (*ε_zz_*) *vs*. load-step; (**b**) average axial stress (*σ_zz_*) *vs.* load-step; (**c**) von Mises stress *vs*. load-step, for the entire domain in the two-grain polycrystal model.

The average axial strain component (*ε_zz_*), the average axial stress component (*σ_zz_*) and the average von Mises stress plotted against the load-step for the entire domain are shown in Figure 4a–c, respectively. The average values of the studied field quantities were calculated by taking the volume-weighted average over all the elements. With regards to the average RUC behavior, the GMC results show excellent agreement with the FEA solutions.

**Figure 5:**
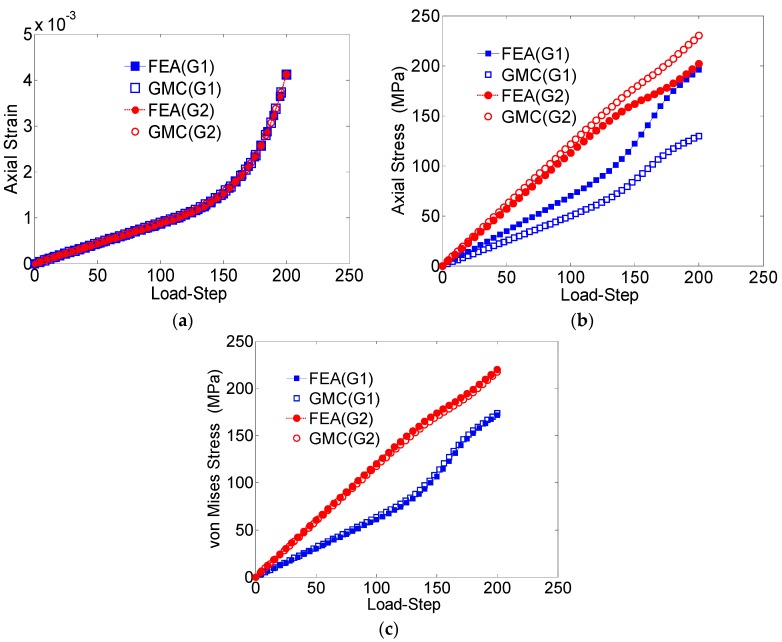
(**a**) Average axial strain (*ε_zz_*) *vs*. load-step; (**b**) average axial stress (*σ_zz_*) *vs*. load-step; (**c**) von Mises stress *vs*. load-step for the individual grains, Grain1 (G1) and Grain 2 (G2).

The local microstructural response of the RUC is also studied in Figure 5 by comparing the GMC *vs.* FEA results with regard to the average element-wise behaviors corresponding to each of the grains for the applied stress load. For the axial stress *vs.* the load step (Figure 5b), some discrepancy between the reported results from GMC and FEA for each individual grain is observed. The difference is significant in the elastic regime, which contributes to the difference in the overall behavior. On the contrary, the average von Mises stress behavior (Figure 5c), which is a measure of the shear deformation, and the average axial strain (Figure 5a) demonstrates good agreement in both the elastic and plastic zones.

**Figure 6:**
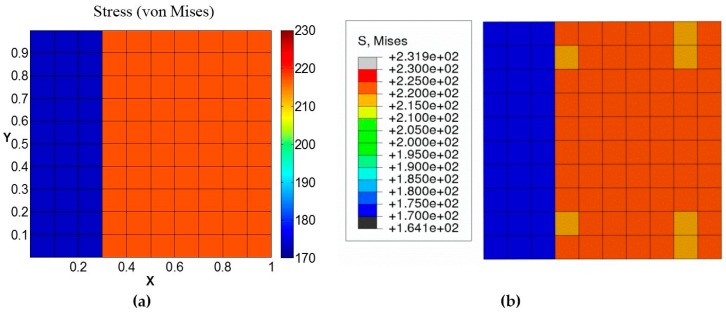
Von Mises stress distribution on a cross-section taken midway through the length in the loading direction for the two-grain polycrystal model by: (**a**) GMC; (**b**) FEA.

Table 2 reports the maximum error percentile for each of these cases. Similarly, the von Mises stress distribution at a cross-section taken midway through the length in the loading direction for the strain loading case also matches in each grain (Figure 6).

Therefore, it can be concluded from these results that GMC could be quite effective for homogenization in terms of predicting the effective stress quantities, such as von Mises, while the prediction of individual components of stress tensor could be below satisfactory due to homogenization and lack of shear coupling.

#### 3.2.2. Voronoi Polycrystal

**Figure 7:**
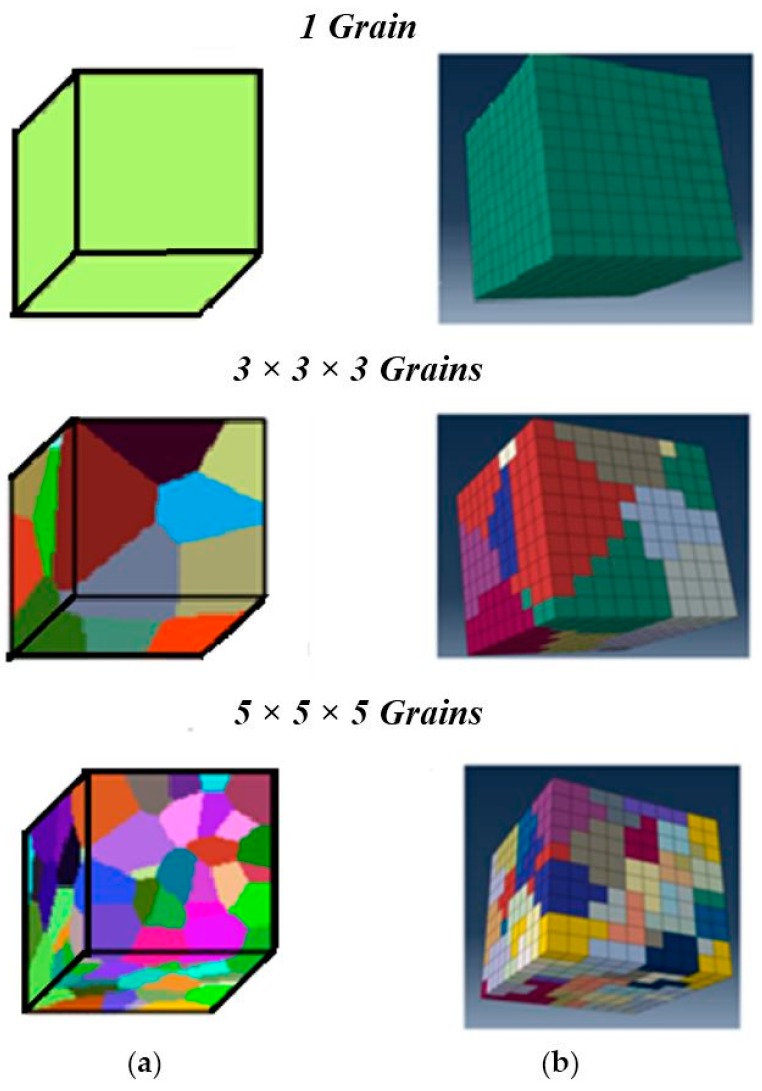
Polycrystal geometry formation through: (**a**) Voronoi tessellations; (**b**) corresponding FEA and GMC discretization.

The GMC integrated with single crystal plasticity constitutive model is further verified by analyzing polycrystalline samples with additional grains and random orientations (Figure 7).

The *n* × *n* × *n* grains terminology refers to *n* grains (roughly) in each dimension with a total of *n*^3^ grains (exact) in the sample. Figure 7a shows the grain geometry generated using Voronoi tessellations in MATLAB, and Figure 7b shows the corresponding finite element and GMC models. The orientations were assigned according to the randomly selected orientation vectors for each of these grains. Overall, there is an agreeable match between the discretized model geometries and the target tessellation geometry. The slight mismatch is mainly because of the fact that the color coding is based on the element centroids for the finite element model, while the MATLAB script follows the exact surface. Hence, this approach of meshing the sample geometry first, followed by assigning the grain orientations to the elements based on their location of centroids rather than meshing the grain structure directly, has an inherent drawback in that it cannot match the grain geometry exactly. However, this can be reduced significantly by refining the mesh sufficiently to make element size much smaller than the grain size.

**Figure 8:**
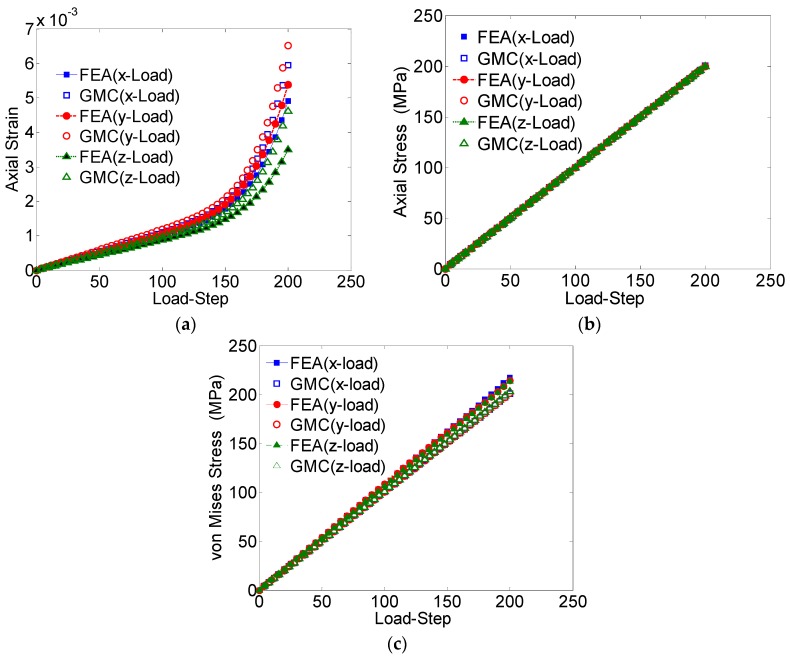
(**a**) Average axial strain (*ε_zz_*) *vs*. load-step; (**b**) average axial stress (*σ_zz_*) *vs*. load-step; (**c**) von Mises stress *vs*. load-step for a 3 × 3 × 3 grain polycrystal (loaded in the three Cartesian coordinate directions).

The GMC and FEA results are compared for the 3 × 3 × 3 grain sample under the uniaxial applied tensile loading (Figure 8).

The average stress-strain behaviors obtained from both these methods, in all the three directions, are shown in Figure 8. It can be observed that for the entire domain, the converged GMC solutions related to the axial stress (Figure 8b) and von Mises stress (Figure 8c) match well with the FEA solutions; however, the axial strain does not show good agreement. The axial strain, especially that obtained by FEA analysis, is a result of the anisotropy in the materials, which can be improved by increasing the number of grains in the sample.

**Figure 9:**
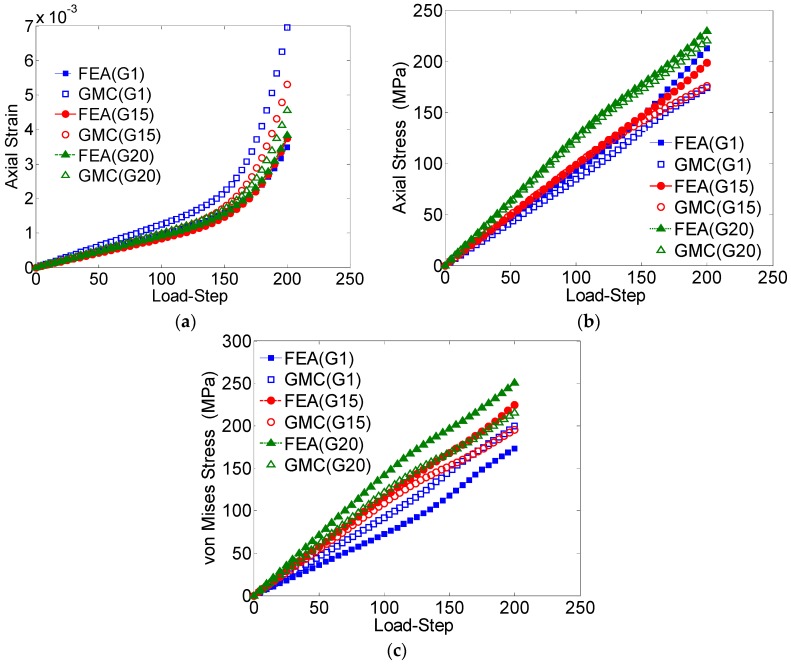
(**a**) Average axial strain (*ε_zz_*) *vs*. load-step; (**b**) average axial stress (*σ_zz_*) *vs*. load-step; (**c**) von Mises stress *vs*. load-step, for three randomly selected grains.

The average values of field quantities in the individual grains, for the case of loading in the *z-*direction, are further studied by comparing the behavior of three arbitrarily chosen grains (Grain numbers 1, 15 and 20) obtained by GMC and FEA (Figure 9).

The axial strain (Figure 9a) shows a relatively large difference, while the axial stress demonstrates excellent agreement. Though the difference was relatively larger for von Mises stress when compared to the axial stress, it was still within a reasonable range: around 15% error after the loading is completed. This difference can be attributed to the lack of normal-shear coupling in the GMC formulation, which introduces error in the stress concentrations near grain boundaries.

**Figure 10:**
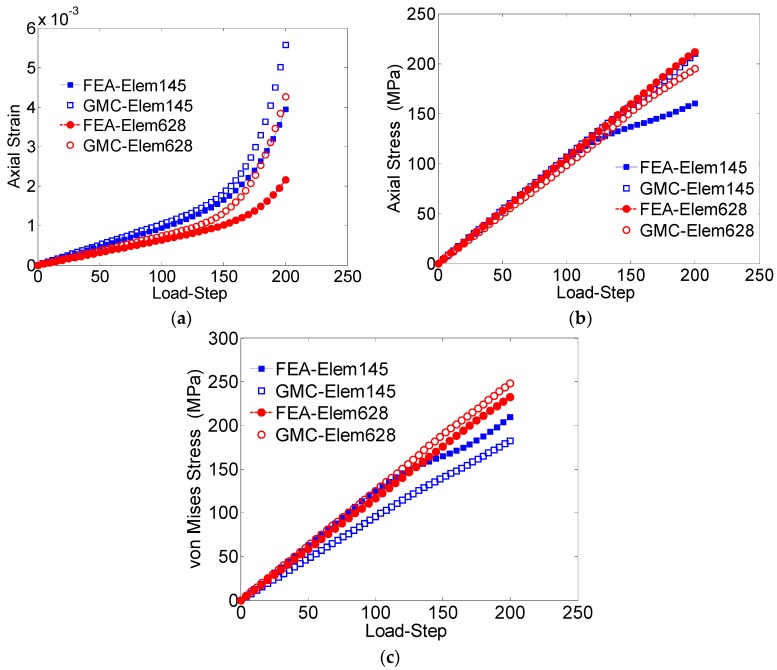
(**a**) Axial strain (*ε_zz_*) *vs*. load-step; (**b**) average axial stress (*σ_zz_*) *vs*. load-step; (**c**) von Mises stress *vs*. load-step, for two randomly selected elements/subcells.

To further study the effectiveness of GMC, the results of two arbitrarily chosen subcells were compared with the corresponding elements in the FEA predictions. One of the studied elements/subcells was located far from the grain boundary (element 628), while the other (element 145) was in the region near boundary between the grains. The GMC and FEA results of the axial strain show significant difference (Figure 10a) in each of the elements. The axial stress results agree closely for element 628, away from the boundary, while they demonstrate significant difference for element 145, which is closer to the boundary (Figure 10b).

In the case of von Mises, element 628 has excellent agreement, while element 145, closer to the grain boundary, shows reasonable agreement. Even in the elastic regime, the GMC and FEA von Mises stresses differ because of additional shear stresses introduced through normal-shear coupling in the FEA model. A more detailed comparison is provided in Table 3, which clearly shows that the GMC solution includes a maximum of 7% error when compared to FEA for the element far away from the grain boundary, but 17% error for the element closer to the grain boundary.

**Figure 11:**
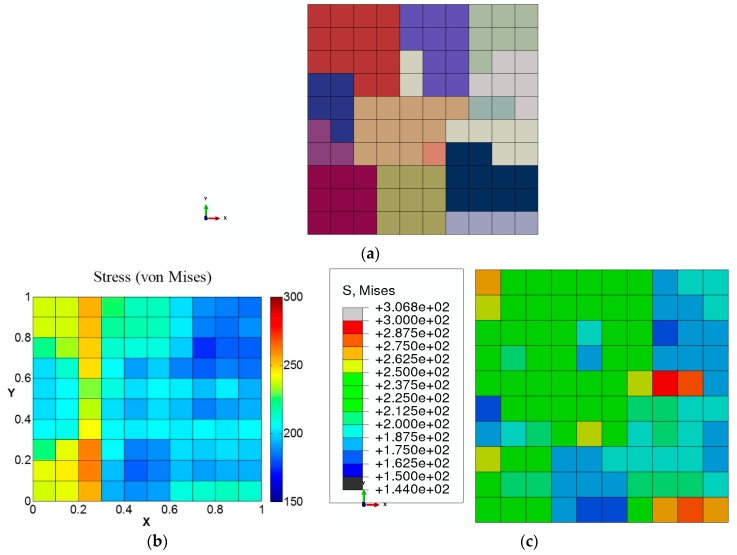
(**a**) Material distribution on the studied cross-section; (**b**) von Mises stress obtained by GMC; (**c**) von Mises stress obtained by FEA.

In addition, the von Mises stress distribution on the cross-section is compared in Figure 11. It can be observed from the comparison that von Mises stress distribution determined by GMC agrees reasonably well with FEA in individual grains, though the gradient near the grain boundaries (as was also concluded from Figure 10) is not captured as well in GMC.

The computational speeds were compared between GMC and FEA for three different cases: Case (1) a single crystal with one element/subcell; Case (2) 27-grain polycrystal with 1000 elements/subcells and Case (3) 125-grain polycrystal with 2744 elements/subcells (Table 4). GMC demonstrated a significant reduction in the computational cost. The computational speeds of GMC were 90, 209 and 239 times faster than that of FEA for Cases 1, Case 2 and Case 3, respectively.

The significant savings in the computational cost in addition to the capability to predict the average RUC field quantities with minimal error make GMC homogenization a reliable and efficient method for analyzing large-size problems. Large stress gradients near the grain boundaries due to the abrupt change in the orientation of the grains adversely impact the local prediction accuracy of GMC due to the lack of normal-shear coupling, especially for the components of field tensor quantities averaged over the grains. However, the error on the prediction of effective quantities such as von Mises is within a reasonable range. The local values of the stress tensor components and the von Mises stress, determined at the element integration points, showed higher accuracy for the elements away from the grain boundary. Though the prediction error of the stress tensor components were relatively high for elements near the grain boundary, the error associated with the von Mises remained within reasonable limits. It is also important to note that the stress gradients near grain boundaries in real materials are not as sharp as that predicted by FEA due to various grain boundary deformation mechanisms and smoother grain boundaries. Hence, difference in the prediction between FEA and GMC decreases in a more realistic case than that observed in the present study. In summary, it can be stated based on the results from the present study that the GMC method can be used for extracting the effective von Mises stress (critical quantity for determining failure in metalic materials) at microscale with a reasonable accuracy, while reducing the computational cost significantly. Moreover, it is the average RUC stresses and strains that provide the link (*i.e.*, “handshake”) between the macro- and microscales. Thus, the good agreement in the prediction of the average RUC fields demonstrates that GMC is a viable subscale tool for integration in a multiscale framework.

### 3.3. Disc

GMC integrated with the single crystal constitutive model is linked to macroscale through an FEA framework (FEAMAC) to develop the multiscale model that can analyze structural scale problems consisting of millions of grains. The macroscale strain, updated in each time-step, is determined at the integration points of the macroscale FEA elements. These integration point quantities are applied to the RUCs (microscale) homogenized by GMC, providing the average stresses, stiffness and inelastic strains back to the FEA (macroscale) integration point. For the purpose of demonstrating this functionality, a segment of a real-size turbine disk is analyzed. The disk is 254 mm long and is exposed to a centrifugal load ($\rho \omega^{2})$ with the magnitude of 6.3 $\frac{\text{kg}\cdot {\text{rad}}^{2}}{{\text{mm}}^{3}\cdot s^{2}}$ (corresponding to the squared angular speed of 7 × 10^8^ ${\left(\frac{\text{rad}}{\sec}\right)}^{2}$). Considering the symmetry of the geometry and the load, 1/8 of the disk is analyzed applying appropriate symmetric boundary conditions and 20-node brick elements. It is important to note that the symmetric assumption is valid strictly only at the macroscale. The violation of this assumption due to the anisotropy and inhomogeneity arising from the differences in grain orientations at microscale does not have any significant impact on the present demonstration study, and is hence ignored. The 3 × 3 × 3-grain polycrystal was used as the material model for each element in the microscale domain.

**Figure 12:**
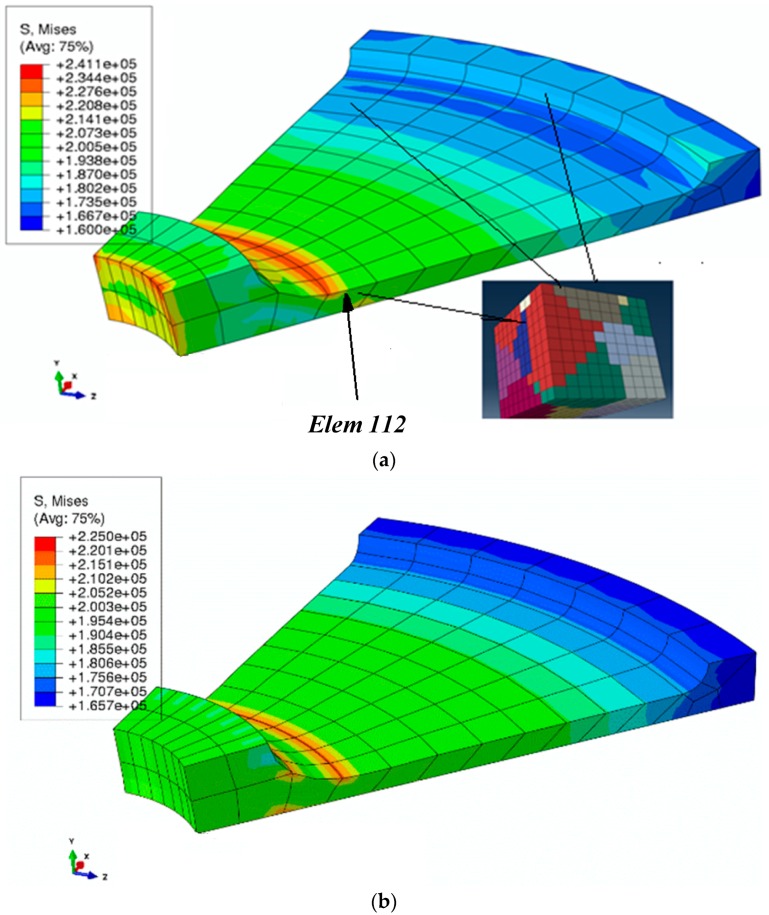
Von Mises stress distribution $\left(\times 10^{-3}\text{ MPa}\right)$ in the real-size disk example: (**a**) linking macroscale to microscale using FEA/GMC; (**b**) applying the standard FEA model with a macroscale constitutive relationship.

Figure 12a shows the von Mises stress distribution in the disk. To compare the macroscale stress distribution, the von Mises stress distribution obtained by using a standard finite element method (single scale) with a macroscale plasticity model is shown in Figure 12b. In the standard FEA model, the global stress–strain curve obtained from the same 27-grain polycrystal under uniaxial loading was used as the constitutive model. It is important to note that the stress–strain variation per element in the standard FEA follows the element interpolation, while in the multiscale model the GMC can evaluate the stress–strain distribution at the microscale consistent with the interpolated macroscale values of the macroscale finite element.

**Figure 13:**
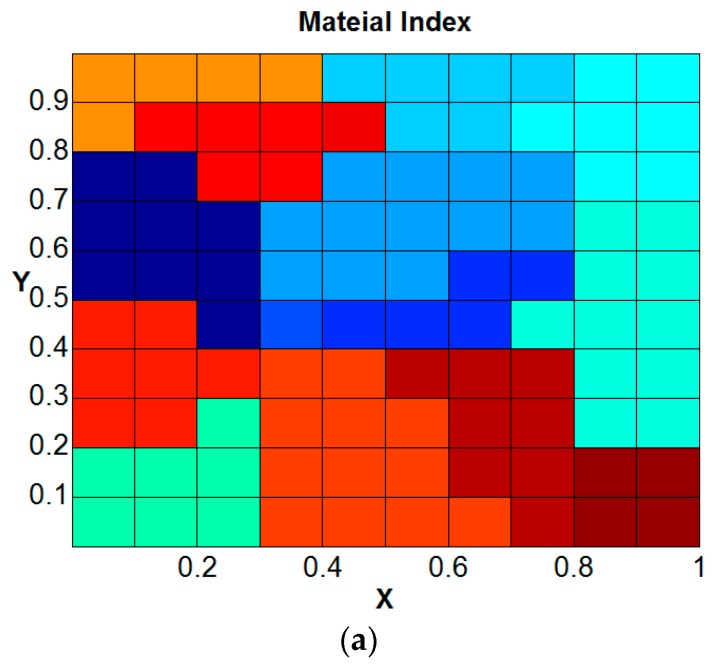

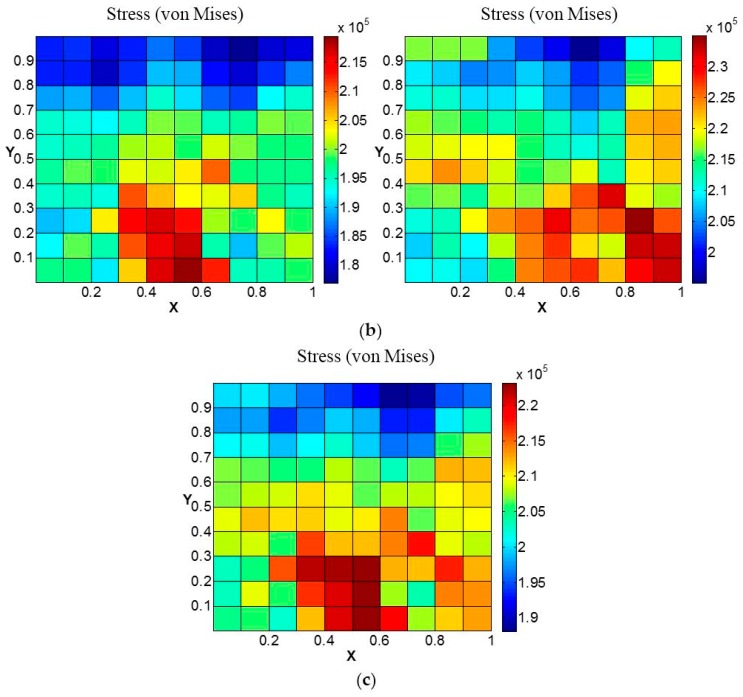
Micro-level study at integration points (for Element 112): (**a**) chosen cross-section; (**b**) von Mises stress in two chosen integration points; (**c**) the average value of von Mises stress over all the integration points of the element.

The von Mises stress distributions shown in Figure 12a,b demonstrate that the multiscale linking using GMC homogenization under a finite element framework predicts expected distribution at macroscale. The cross-sectional von Mises stress distribution for an element (Element 112), chosen arbitrarily, is shown in Figure 13, and the element location is indicated in the model in Figure 12a).

The distribution of the grain orientations on the chosen cross-section of the RUC and the von Mises stress distribution on this cross-section for two of the integration points of a random element (here Element 112) are demonstrated in Figure 13a,b, respectively. In addition, the average von Mises stress distribution for all the integration points chosen on the previously defined RUC cross-section is shown in Figure 13c. The results show that the local von Mises distribution at the grain-level in a real-sized model can be determined using the developed multiscale model, which is extremely computationally expensive, with a standard FEA approach.

## 4. Conclusions

A multiscale computational model was developed by employing the single crystal plasticity constitutive model at the microscale, in conjunction with GMC for homogenization, coupled to an FEA framework at the macroscale. In order to verify the effectiveness of GMC as a homogenization tool, the microscale behavior of single and polycrystalline samples were determined using the stand-alone GMC, and compared to that obtained from the standard FEA utilizing higher-order elements. For the polycrystals consisting of tens to hundreds of grains, GMC analysis achieved two–three orders of savings in computational cost at a minimal expense of accuracy in the components of both average and local tensor field quantities. The results based on GMC homogenization demonstrated reasonably good agreement with the FEA solution in terms of the von Mises and stress tensor components for the entire studied polycrystalline domain, the grain-averaged von Mises, von Mises and stress tensor components for elements away from the grain boundaries and von Mises for elements near the grain boundary. Therefore, the von Mises stress was found to be in a reasonably good agreement for all cases, making GMC a promising method for failure analysis applications.

Although the large gradient near the grain boundaries was not captured by GMC, the applicability of GMC homogenization is expected to be more effective in real materials where behavior does not vary at the grain boundaries as drastically as in the model due to various grain boundary deformation mechanisms. In addition, the accuracy of the local fields may not be pertinent in the absence of failure localization, as long as the average stress and strain quantities are accurate, since this is the mode for transmitting information across the scales in this multiscale framework.

Finally, the multiscale aspect of the model was demonstrated by implementing GMC as a homogenization tool on an FEA platform and investigating a real life turbine engine disc problem. The macroscale results demonstrated the expected stress distribution when compared to the FEA-based analysis, thereby verifying the method. The microscale distribution of von Mises stress was extracted on the cross-section of an arbitrarily selected element. The results demonstrated the multiscale capability of the developed multiscale model and may allow engineers to model variability in the microstructure spatially within a structural component and tailor the microstructure for different structural applications.

## Figures and Tables

**Table 1:** Grain data used for studying the simple polycrystal case.

Grain #	Volume Fraction	1st Vector		2nd Vector	
		Local	Global	Local	Global
1	30%	(0,0,1)	(0,0,1)	(1,0,0)	(1,0,0)
2	70%	(1,1,0)	(0,0,1)	(1,−1,0)	(1,0,0)

**Table 2:** Maximum percentile error of the GMC solution (compared to FEA) for the two-grain polycrystal sample.

Domain	Average Axial Strain Error %	Average Axial Stress Error %	Average
Entire Domain	−0.231	0.036	−0.706
Grain 1	−0.232	−33.891	1.146
Grain 2	−0.232	14.131	−1.325

**Table 3:** Maximum percentile error of the GMC solution (compared to FEA) for the 3 × 3 × 3 polycrystal sample.

Domain	Average Axial Strain Error %	Average Axial Stress Error %	Average von Mises Error %
Entire Domain	37.338	0	−5.650
Grain 1	99.575	−18.245	15.482
Grain 15	41.738	−11.280	−12.998
Grain 20	18.648	−3.996	−13.992
Element 145	41.343	30.952	−17.062
Element 628	97.511	−8.633	6.932

**Table 4:** Comparison of computational time between FEA and GMC models.

# of Grains	FEA/Abaqus	MAC/GMC	MAC/GMC Speedup Per Iteration
1 grain/1 element	12 s 0.021 s/iter	0.06 s 0.000235 s/iter	90×
3 × 3 × 3 = 27 grains/1000 elements	297 s 4.18 s/iter	5.062 s 0.02 s/iter	209×
5 × 5 × 5 = 125 grains/2744 elements	1438 s 16.72 s/iter	18.63 s 0.07 s/iter	239×
